# Oligometastatic Mesothelioma Treated with Ablative Radiotherapy (OMAR): A Multicenter Study

**DOI:** 10.3390/cancers17172797

**Published:** 2025-08-27

**Authors:** Davide Franceschini, Paolo Ghirardelli, Patricia Frrokaj, Nicolaus H. Andratschke, Luca Nicosia, Elisabetta Parisi, Gaia Piperno, Matteo Sepulcri, Emanuele Alì, Antonio Marco Marzo, Stefano Bendoni, Ruggero Spoto, Marco Krengli, Patrizia Ciammella, Barbara A. Jereczek-Fossa, Antonino Romeo, Rosario Mazzola, Filippo Alongi, Matthias Guckenberger, Giovanni Luca Ceresoli, Mauro Loi, Paolo Borghetti, Marta Scorsetti

**Affiliations:** 1 Department of Radiotherapy and Radiosurgery, IRCCS Humanitas Research Hospital, 20089 Rozzano, Italy; 2 Department of Radiotherapy, Cliniche Humanitas Gavazzeni, via Gavazzeni 21, 24125 Bergamo, Italy; 3 Department of Radiation Oncology, University Hospital Zurich, University of Zurich, Rämistrasse 100, 8091 Zurich, Switzerland; 4 Advanced Radiation Oncology Department, IRCCS Sacro Cuore Don Calabria Hospital, Cancer Care Center, 37034 Negrar Di Valpolicella, Italy; 5 IRCCS Istituto Romagnolo per lo Studio dei Tumori (IRST) “Dino Amadori”, 47014 Meldola, Italy; 6 Division of Radiotherapy, IEO European Institute of Oncology IRCCS, 20141 Milan, Italy; 7 Radiation Therapy Unit, Veneto Institute of Oncology IOV-IRCCS, 35128 Padova, Italy; 8 Radiation Oncology Unit, Azienda USL-IRCCS di Reggio Emilia, 42123 Reggio Emilia, Italy; 9 Dipartimento di Scienze Chirurgiche, Oncologiche e Gastroenterologiche, Università di Padova e Istituto Oncologico Veneto—IRCCS, 35128 Padova, Italy; 10 Department of Oncology and Hemato-Oncology, University of Milan, 20122 Milan, Italy; 11 Department of Biomedical Sciences, Humanitas University, 20090 Pieve Emanuele, Italy; 12 University of Brescia, 25121 Brescia, Italy; 13 Department of Medical Oncology, Cliniche Humanitas Gavazzeni, via Gavazzeni 21, 24125 Bergamo, Italy; 14 Department of Radiation Oncology, Azienda Universitaria Ospedaliera Careggi, 50134 Florence, Italy; 15 Radiation Oncology Department, ASST Spedali Civili and University of Brescia, 25123 Brescia, Italy

**Keywords:** pleural mesothelioma, oligometastases, oligorecurrence, stereotactic ablative radiation therapy

## Abstract

Background/Objectives: This multicenter retrospective study aims to evaluate the role of Ablative Radiotherapy (RT) in patients with unresectable pleural mesothelioma (PM) who experienced radiological progression after at least one line of chemotherapy, with a maximum involvement of three pleural or extrapleural sites. Methods: Adult patients (≥18 years) with PM treated with stereotactic radiotherapy between 2011 and 2022, limited to a maximum of three pleural or extrapleural sites, were included in the analysis. Ablative RT was required to be administered with radical intent. Endpoints were time to further systemic therapy (TFST), local control (LC), progression-free survival (PFS), overall survival (OS), and acute and late radiotherapy-related toxicity. Results: A total of 56 patients were identified from six Italian and one Swiss radiotherapy center. Treatment was generally well tolerated. Ten patients experienced grade 1 or 2 acute toxicity, while four patients reported persistent chest pain, with one case reaching grade 3 as late toxicity. The median TFST was 18.6 months, with TFST rates of 61.7% and 46.4% at 12 and 24 months, respectively. The median OS was 37.63 months, with 1- and 2-year OS rates of 85.2% and 65.6%. Local control was favorable (79% at 1 year), but most patients experienced disease recurrence outside the SABR treatment volume. The median disease progression-free survival (DPFS) was 8.17 months, with 1- and 2-year DPFS rates of 36% and 19%, respectively. Smoking history correlated with OS and DPFS in univariate analysis, while statistical significance for OS was maintained in multivariate analysis. Additionally, nodal status and PTV volume were associated with OS. Conclusion: SABR is a safe and effective approach for the treatment of oligorecurrent/oligoprogressive PM. The time to further systemic therapy was extended up to 18 months. At two years, 10% of patients remained disease-free, and more than half were alive at three years, suggesting a potentially indolent biological behavior in oligometastatic PM.

## Simple Summary

The safety and efficacy of Ablative Radiotherapy (RT) in oligorecurrent/oligoprogressive pleural mesothelioma (PM) were evaluated in this multicenter retrospective study. A total of 56 patients, corresponding to 82 treated lesions, were included in the analysis. The median time to further systemic therapy was extended up to 18 months, while the median overall survival (OS) exceeded 3 years. No significant side effects were observed, except for a single case of grade 3 late toxicity. SABR proved to be an effective and safe treatment in this selected cohort of patients.

## 1. Introduction

Pleural mesothelioma (PM) is an aggressive malignancy originating from the pleural lining and is most commonly associated with asbestos exposure [1]. Despite recent advances in multimodal therapy—combining chemotherapy and/or radiotherapy with mesothelioma resection—the median survival remains limited to approximately 23 months [2]. Moreover, only a minority of patients are eligible for multimodal therapy, including surgery (MMT), as the majority are diagnosed with diffuse disease and can therefore receive systemic treatment only. Despite advances in the understanding of the biology and natural history of pleural mesothelioma, effective treatment options remain lacking for patients who experience progression during or after first-line therapy [3,4,5]. New therapeutic strategies are urgently needed.

Radiotherapy (RT) has traditionally been employed in patients with PM, mainly for the palliative management of chest wall pain [6] or as a component of multimodal therapy (MMT) in early-stage disease [7,8]. However, recent studies on various solid tumors have validated a new role for RT in managing so-called “oligometastatic disease” [9,10,11,12].

Applying these concepts to PM remains challenging due to the disease’s unique characteristics and behavior. However, in clinical practice, isolated local recurrences, progression, or persistence of disease can be observed, a phenomenon that may become even more frequent with recent advancements in systemic therapies. In these selected cases, investigating the potential role of local treatments is an intriguing prospect.

An expert consensus supports Stereotactic Ablative Radiotherapy (SABR) as a safe treatment, recommending high-dose per-fraction delivery for localized or isolated PM recurrences to achieve relatively high long-term local control (LC) rates [13]. SABR offers a promising approach for these patients, delivering a biologically ablative radiation dose with low toxicity. However, studies investigating SABR for relapsing PM remain scarce [14,15,16,17,18]. Globally, these four studies included 117 patients, the largest experience being reported by Shin et al. (44 patients). All these studies took similar conclusions from their own data analysis: radiotherapy is safe, can obtain durable local control and delay the need for further systemic therapy. At the same time, all authors highlighted how these results should be interpreted with caution, due to the low number of patients and the heterogeneity of a retrospective analysis.

This multicenter retrospective study aims to evaluate whether ablative RT can influence the disease course in patients with unresectable PM presenting with radiological oligorecurrence, oligoprogression, or oligopersistence after at least one line of chemotherapy, with a maximum of three pleural (or extrapleural) sites involved.

## 2. Material and Methods

This study identified adult patients with PM who were treated between 2011 and 2022 with SABR for a maximum of three pleural or extrapleural sites. Given the aggressive nature of PM, a stricter definition was applied to classify patients as oligometastatic (OM). Patients were categorized according to the ESTRO-EORTC consensus [19], and those with oligorecurrent, oligopersistent, or oligoprogressive disease were included in the trial.

Inclusion criteria were as follows:

- Histological diagnosis of PM;
- At least one line of systemic therapy already received;
- Unresectable disease;
- Number of lesions ≤ 3 in pleural or extrapleural sites;
- RT delivered with ablative purposes (50 Gy EQD2/10 delivered in a maximum of 12 fractions as per Oligocare definition—NCT03818503);
- Ability to provide written informed consent.

Demographic, clinical, and treatment data were collected.

Clinical and therapeutic variables analyzed were age, sex, performance status at diagnosis, smoking habit, PM histology and staging, treatment received for primary diagnosis, disease-free interval, oligometastatic classification, previous local and systemic therapies, RT dose and fractionation, Biologically Effective Dose (BED), number and site of irradiated lesions, subsequent therapies for further disease progression.

The analysis was first approved by the ethical committee of the Humanitas Research Hospital, and subsequently by all the participant centers.

Endpoints of the present analysis were as follows:

- Time to further systemic therapy (TFST): defined as the time from the first day of RT to the start of any further systemic therapy, if delivered; if not, until the day of disease progression or death, whichever occurred first.
- Local control (LC): defined as the absence of progression within the volume of RT, assessed by contrast-enhanced CT scan and or FDG-PET.
- Distant progression-free survival (DPFS): time from the first day of RT until the date of detection of progressive disease (out-field) or death, whichever occurred first.
- Progression-free survival (PFS): time from the first day of RT until the date of detection of progressive disease (in-field or out-field) or death, whichever occurred first.
- Overall survival (OS): time from the first day of RT to death. Patients lost to follow-up had their OS censored at the last date they were known to be alive.
- Acute and late toxicities according to CTCAE v 5.

Patients were followed up after treatment as per institutional protocol and general guidelines. Generally, patients underwent CT 3 months after RT and PET FDG 6 months after.

Progressive disease was defined according to the modified RECIST criteria for PM [20,21].

### Statistical Analysis

Continuous variables are reported as median values with interquartile ranges, while categorical variables are expressed as percentages. Survival analyses were performed using non-parametric Kaplan–Meier curves. Potential risk factors were evaluated using univariate Cox regression, with Hazard Ratios (HR) and 95% confidence intervals (CI) reported for each variable. Multivariate analysis was conducted for variables that were statistically significant (*p* < 0.05).

All statistical analyses were performed using STATA, version 18.

## 3. Results

A total of 56 patients (82 treated lesions) were enrolled in this study from six Italian and one Swiss center. Patient demographics, treatment details, and tumor characteristics are summarized in Table 1 and Table 2.

The majority of patients were male and either current or former smokers. Epithelioid histology was the most prevalent subtype. More than half of the patients underwent surgery as the primary treatment for their PM diagnosis, though only nine received adjuvant radiotherapy to the pleural cavity (25 Gy in 5 fractions in four patients, 44 Gy in 22 fractions in one patient and 50 Gy in 25 fractions in four patients). Disease recurrence occurred within one year of the initial diagnosis in most cases, with a median disease-free interval of 11.3 months (range 6.6–137.7 months). In five patients relapse occurred in the field of previous adjuvant RT. The majority of patients received RT for an “oligo” diagnosis after their first line of systemic therapy. Approximately 60% of patients underwent irradiation at a single site, with varying doses and fractionation schedules (minimum 36 Gy in six fractions, maximum 55 Gy in five fractions). The median Biological Effective Dose (BED), assuming an alpha/beta ratio of 10 Gy, was 61.25 Gy (range 50–115.5 Gy).

No acute toxicity higher than grade 2 was observed within three months of RT. The most commonly reported side effect was chest wall pain (grade 1 in four patients, grade 2 in two patients). Additional side effects included cough (grade 1 in one patient, grade 2 in one patient), grade 2 esophagitis (one patient), and grade 1 dyspnea exacerbation (one patient). Late toxicity included persistent chest wall pain in four patients (two grade 1, one grade 2, and one grade 3). No additional toxicity was recorded in patients previously treated with adjuvant RT.

The median follow-up was 27.8 months (range 2.2–116.9 months).

During follow-up, 32 patients (57.1%) initiated a new systemic therapy. Among the remaining patients, 19 (33.9%) experienced disease progression but did not start a new active treatment due to poor general condition. Only five patients remained free from both local and distant progression.

**Figure 1:**
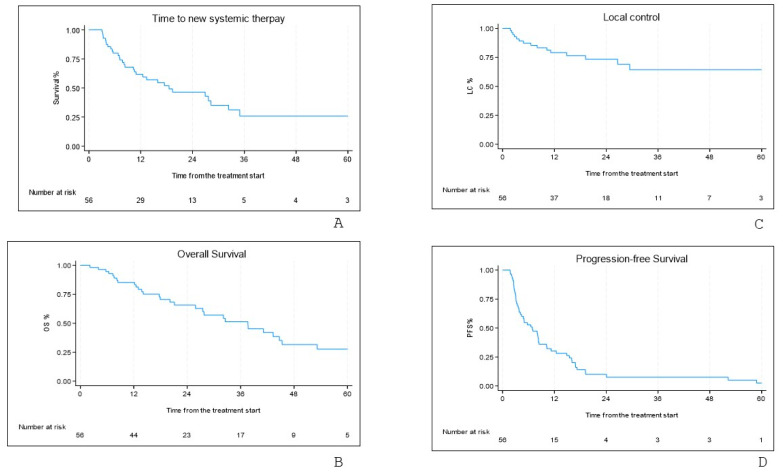
Survival curves: time to further systemic therapy-free survival (**A**), overall survival (**B**), local control (**C**), progression-free survival (**D**).

The median TFST was 18.6 months (IQR 7.2–63.8 months). TFST rates at 12 and 24 months were 61.7% and 46.4%, respectively (Figure 1A). Univariate analysis showed no significant correlation between TFST and any analyzed variable.

At the last follow-up, 24 patients (42.8%) were alive, while 32 (57.2%) had died due to disease progression. The median OS was 37.6 months (IQR 17.7–61.7 months), with 12- and 24-month OS rates of 85.2% and 65.6%, respectively (Figure 1B).

**Figure 2:**
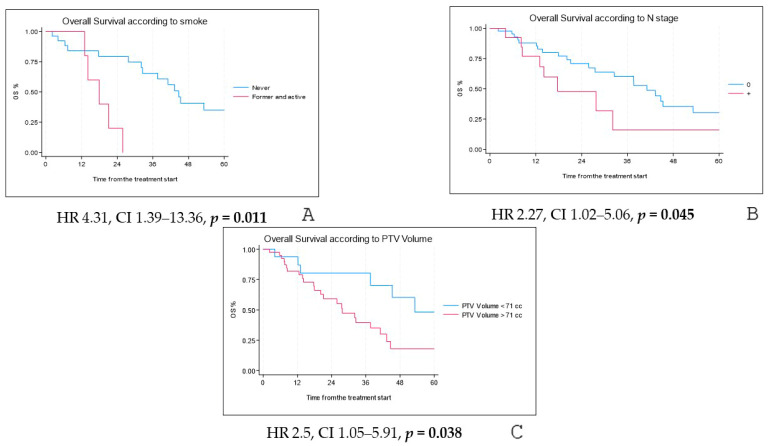
Overall survival according to smoking status (**A**), N stage at diagnosis (**B**) and PTV volume (**C**).

Univariate analysis revealed that never-smokers had significantly longer OS (HR 4.31, CI 1.39–13.36, *p* = 0.011). Nodal status at diagnosis and PTV volume also correlated with OS (HR 2.269, CI 1.018–5.062, *p* = 0.045 and HR 2.496, CI 1.054–5.912, *p* = 0.038, respectively) (Figure 2A–C). However, in multivariate analysis, only smoking history remained significantly associated with OS (HR 3.706, CI 1.192–11.524, *p* = 0.024).

**Figure 3:**
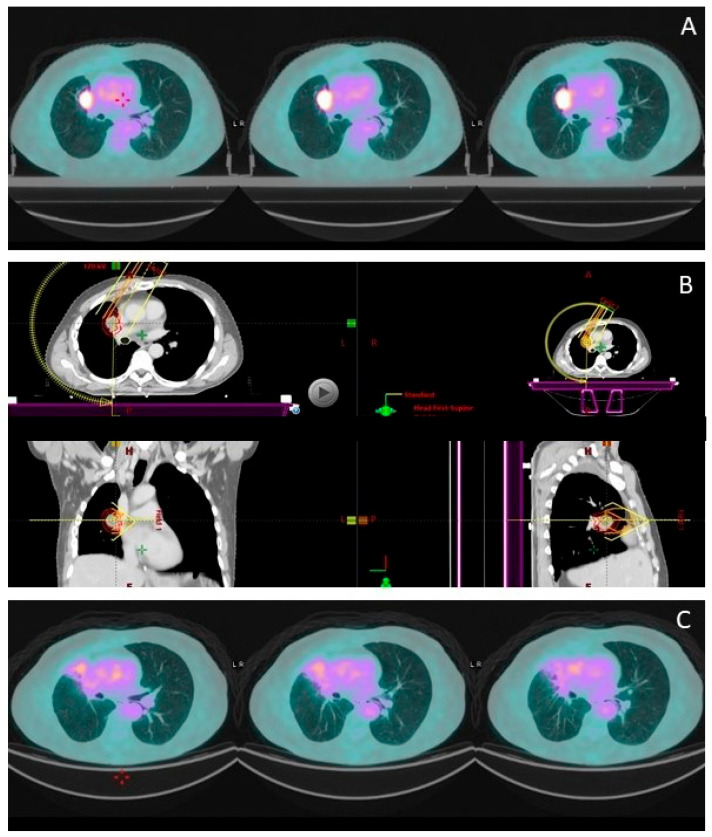
An example of a pleural relapse (**A**) treated with SABR 50 Gy in five fractions (**B**) with complete response at follow-up (**C**).

Local relapse occurred in 15 patients (26.8%). The median time to local control (LC) was not reached (IQR 19.23 months—not reached). LC rates at 12 and 24 months were 79% and 73%, respectively (Figure 1C). No analyzed variables showed a significant correlation with LC. An example of local response is shown in Figure 3.

**Figure 4:**
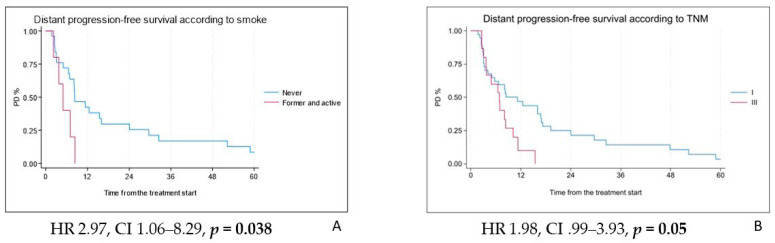
Distant progression-free survival according to smoking status (**A**) and TNM stage at diagnosis (**B**).

Most patients (48, 84.2%) experienced progression outside the RT field, with the pleura being the most common site of relapse (58.3%). The median distant progression-free survival (DPFS) was 8.2 months (IQR 3.4–16.9 months), with 12- and 24-month DPFS rates of 36% and 19%, respectively. Univariate analysis identified smoking history and TNM stage (stage I vs. stage II or III) as significant predictors of DPFS (HR 2.969, CI 1.064–8.287, *p* = 0.038 and HR 1.982, CI 0.999–3.934, *p* = 0.05, respectively) (Figure 4A,B).

Considering both local and distant relapse, the median PFS was 6.9 months (IQR 3.06–15.47 months). PFS rates at 12 and 24 months were 30% and 10%, respectively (Figure 1D). Univariate analysis showed no significant correlation between PFS and any analyzed variable.

## 4. Discussion

To the best of our knowledge, this is the largest series analyzing the outcomes of ablative intent RT for oligometastatic PM.

The results presented highlight the feasibility of this approach, demonstrating a significant impact on the natural history of the disease. Specifically, we observed a median TFST of 18 months, a notable improvement compared to previously published experiences. For instance, in a prior study involving two of the institutions that participated in the OMAR study, the median TFST was only six months [14]. Barsky et al. [16] reported a longer median TFST of 10.9 months in a retrospective analysis of 15 patients with PM treated with SABR on 25 lesions.

Patient selection and the larger sample size in our study may explain these results. Most of our patients received irradiation during or after the first line of systemic therapy and were therefore not heavily pretreated. In contrast, in the study by Ghirardelli et al. [14], 38% of patients had already received two or more lines of systemic therapy. Furthermore, more than half of our patients were treated for oligopersistence or oligorecurrence, whereas other studies focused exclusively on oligoprogression, a condition more prone to rapid disease progression and requiring a change in systemic therapy [22,23].

Our analysis found a higher-than-expected local recurrence rate. LC at one and two years was 79% and 73%, respectively, lower than the commonly reported LC rates in SABR studies, which typically range between 80% and 90% [24,25,26]. We could not identify any parameters associated with local recurrence. The median BED (61.25 Gy) in our study was lower than the standard BED used in SABR (typically around 100 Gy). Additionally, reports suggest that PM exhibits intrinsic radio resistance, which may necessitate higher doses per fraction to achieve optimal control [27,28]. However, we found no correlation between BED, dose, or fractionation and the risk of local relapse.

Comparing our results with similar studies in the literature reveals variable outcomes. Our LC rates are consistent with those reported by Ghirardelli et al. (76% at one year) [14] and Schroder et al. (73.5% at one year) [15]. However, Barsky et al. [16] and Shin et al. [17] reported excellent LC rates (100% and 92.9% at one year, respectively) in their studies on 15 and 44 patients. These discrepancies are likely due to the retrospective nature of these studies, reflecting case selection, variations in treatment techniques, RT prescriptions, and institutional practices over extended timeframes. The debate on the optimal BED for controlling PM lesions remains unresolved. Ghirardelli et al. [14] reported improved LC with BEDs above 100 Gy (considering an alpha/beta ratio of 1.5 and 3), whereas Shin et al. achieved good outcomes with lower BEDs (48 Gy in most cases) [17]. Barsky et al. [16] used a standardized fractionation schedule of 40 Gy in five fractions, with no local recurrences within the first three years. In 2024, Ghirardelli et al. [18] further analyzed patients treated with intermediate or high doses (30–36 Gy in five to six fractions vs. 45–50 Gy in four to eight fractions), reporting 100% six-month and one-year LC in both subgroups.

Since this was a multicenter retrospective study, dose selection was performed on an individual basis by the treating physician, with the aim of balancing potential risks and therapeutic benefits. The inclusion criteria mandated the use of an ablative dose, as defined by the Oligocare criteria. This approach was adopted to minimize dose heterogeneity and to restrict the analysis to patients treated with radical intent through a “stereotactic-like” strategy.

As expected, most patients eventually experienced out-of-field progression. The median DPFS in our study was slightly over eight months and was influenced by smoking habits and TNM stage at diagnosis. When considering both local and distant recurrence, the median PFS dropped to 6.8 months. Interestingly, approximately 10% of patients remained free from disease progression for more than two years, a result comparable to that reported by Schroder et al. [15].

The median OS in our series was nearly 38 months, the longest reported to date. Ghirardelli et al. found similar OS in a selected population treated with ablative doses [18]. This underscores the importance of patient selection in evaluating OS outcomes in oligometastatic patients. Studies with less stringent selection criteria tend to report lower OS. For instance, Shin et al. [17] treated 44 PM patients with SABR without specific selection based on RT doses or prior treatments. Despite achieving higher LC rates, the one-year OS in their cohort was only 36%, likely influenced by performance status. Similarly, studies by Ghirardelli et al. and Schroder et al. [14,15], which also had open selection criteria, reported median OS ranging from 26 to 29 months. By selecting only patients treated with ablative doses and not exclusively for oligoprogression, our study likely included a population with better prognosis, resulting in longer OS.

Smoking history statistically correlated with OS in our study. This could seem unexpected at first glance, considering that smoke is not a major promoter of PM. However, the negative impact of smoke on prognosis and treatment response is in line with the results presented in the meta-analysis by Schaefers et al. [29]. Evaluating data from more than 30,000 patients with different tumor types, the authors showed how active smoking during cancer treatment is an independent adverse prognostic factor, while smoking cessation can result in similar outcomes compared to never-smokers.

Tolerance to RT was generally good, in line with the existing literature. We reported only one case of grade 3 late toxicity, similar to Schroder et al. [15]. Acute toxicity was also well tolerated, with no grade 3 or higher events. Comparable findings have been reported in studies by Shin and Barsky [16,17].

This study has several limitations that should be acknowledged to ensure proper interpretation of the results. OMAR remains a retrospective study and is therefore subject to inherent biases. Patients were well-selected, as most of them were affected by epithelioid histology, were stage I or II at diagnosis, were treated “early” in their medical history, etc. Additionally, there was significant heterogeneity within our cohort, as patients were treated at seven different institutions over a period exceeding ten years. Consequently, variations in doses, fractionation schedules, RT techniques, imaging modalities and timing, systemic therapy use are present.

However, the sample size of our study is a notable strength. Despite the rarity of PM, this topic may become increasingly relevant in the coming years with advancements in systemic therapy [30,31].

The introduction of immunotherapy in clinical practice may help identify a subset of patients with prolonged survival and indolent disease behavior [32]. This subgroup is likely to benefit the most from local approaches such as SABR. Further research is warranted to explore the integration of RT with immunotherapy in PM. A Phase I trial combining SABR with avelumab demonstrated good tolerance and promising LC results, which require validation in subsequent studies [33].

## 5. Conclusions

The present study demonstrates that ablative intent RT is well tolerated and offers favorable outcomes in oligometastatic PM. In selected patients, local treatment seems to influence the disease’s natural history, prolonging the time before initiating or changing systemic therapy. Even in a small subset of patients, SABR can maintain disease-free status for up to two years or longer.

Further studies are needed to determine the optimal RT doses for improving patient outcomes and to investigate the integration of RT with novel systemic approaches for PM.

## Figures and Tables

**Table 1:** Patient, tumor and treatment characteristics.

**Patient and primary disease treatment**		
Variable	Number	Percentage
Sex:		
male	39	69.6%
female	17	30.4%
Age (median/range)	68.5 years (range 38–86; IQ range 62.5–73)	
Smoking status:		
Never	26	46.4%
Former	25	44.6%
Active	5	9%
Histology:		
Epithelioid	50	89.3%
Biphasic	1	1.7%
Sarcomatoid	5	9%
Clinical TNM staging:		
Ia	13	23.2%
Ib	25	44.6%
II	2	3.6%
IIIa	2	3.6%
IIIb	14	25%
Surgery:		
Pleurectomy	31	55.4%
Extrapleural pneumonectomy	3	5.3%
No	22	39.3%
Chemotherapy:		
Neoadjuvant	27	48.2%
Adjuvant	6	10.7%
First line	23	41.1%
Adjuvant RT:		
Yes	9	16%
No	47	84%
**Metastatic disease characteristics**		
Disease-Free Interval (median/range)	11.3 months (range 6.6–137.7; IQ range 6.63–30.93)	
Oligometastasis classification:		
Oligorecurrence/oligopersistence	32	57.1%
Oligroprogression	24	42.9%
Relapse timing:		
during/after first line CT	45	80.4%
After second/third line CT	11	19.6%
**SBRT**		
Number of irradiated lesion(s):		
1	34	60.7%
2	18	32.1%
3	4	7.2%
Irradiated site(s):		
Pleura	40	71.4%
Extrapleural	16	28.6%
PTV volume (cc)	Median 71.3 cc (range 14.4–332.5; IQ range 31.8–113.05)	
BED (median/range)	61.25 Gy (range 50–115.5; IQ range 59.24–100)	
BED Biologically Effective Dose		

**Table 2:** RT dose, number of fractions and BED received by patients in OMAR study.

RT Dose	Number of Fractions	BED	Number of Patients
36	6	57.6	2
35	5	59.5	18
30	3	60	4
37.5	6	60.26	2
42.5	10	60.56	1
37.5	5	61.25	3
40	5	72	3
50	10	75	1
48	8	76.8	1
45	6	78.75	3
49	7	83.3	1
60	10	96	2
50	5	100	8
60	8	105	2
48	4	105.6	4
55	5	115.5	1

## Data Availability

Research data are stored in an institutional repository and will be shared upon request to the corresponding author.
